# A Canine's Behavior and Cognitive State as It Relates to Immobility and the Success of Physical Rehabilitation in the Non-ambulatory Spinal Cord Patient

**DOI:** 10.3389/fvets.2021.599320

**Published:** 2021-09-01

**Authors:** Stephanie A. Thomovsky, Niwako Ogata

**Affiliations:** Department of Veterinary Clinical Sciences, Purdue University, West Lafayette, IN, United States

**Keywords:** physical rehabilitation, neurology, neurosurgery, spinal cord injury, canine, behavior and cognition

## Abstract

Physical rehabilitation (PR) is recommended following spinal cord injury to help improve and maintain muscle elasticity, joint mobility, and nerve health. It can also be used to relieve pain and improve cardiopulmonary fitness in an immobile patient. There is evidence, in human medicine, that PR plays a critical role in mental health and the psychological state of the patient. As part of the assessment phase, human physical therapists often identify psychosocial symptoms and barriers at the start of PR that ultimately may affect improvement in human patients suffering from injury and the loss of mobility. Patient psychological state plays an integral role in healing and outcome during treatment and rehabilitation. Specific interventions set to address these symptoms can better outcome. Arguably, one of the most emotionally traumatizing injuries suffered by a canine patient can be immobility secondary to spinal cord injury. Poorly understood is the role the canine cognitive state plays in the success of rehabilitation following spinal cord injury. Should breed, age, sex, physical fitness, personality, previous experiences and history or home lifestyle, affect the PR assessment of these patients? Do these factors affect eventual outcome following PR? The purpose of this manuscript is to explore psychosocial barriers encountered during injury rehabilitation in humans and determine if the similar barriers need to be considered when assessing a canine patient for spinal cord injury rehabilitation.

## Introduction

Physical rehabilitation (PR) is gaining more and more popularity in veterinary medicine. In the past 20 years, the number of clinics in the United States of America actively offering and performing PR continues to grow. In 2014, there were over 110 facilities in the United States offering physical rehabilitation services to their veterinary clients (1). A major group of canine patients for whom PR is considered a standard of care is the veterinary spinal cord injury patient. Spinal cord injuries are common, accounting for 2% of the total cases presenting to veterinarians^1^. Dogs present with a variety of surgical diseases (intervertebral disc disease, spinal fractures and subluxations, lumbosacral stenosis, and caudal cervical spondylomyelopathy) in addition to non-surgical neurologic diseases (fibrocartilaginous embolism, acute non-compressive nucleus pulposus extrusion and spinal contusion) all of which lead to either complete or partial immobility. PR is often a treatment recommendation in all of these patients (2–4). The focus of rehabilitation in the recumbent spinal cord patient is alleviating pain, improving voluntary motor function, coordination and strength. Consideration is given to spinal stability, muscle tone and voluntary motion when designing a specific PR plan for these patients. There are increasing numbers of veterinary PR papers written on the use of physical rehabilitation in dogs with spinal cord injury and disease (5–8). The focus of these manuscripts are often neurologic-outcome based. Little to no information exists exploring the psychological condition of the canine patient, including personality, cognitive state and motivation, and how this condition influences the PR assessment plan and ultimately the success of physical rehabilitation in these spinal cord injury patients.

## Biopsychosocial Perspective in Human Physical Therapy

It is well-recognized in human medicine that treatment of injury is influenced by emotional, behavioral and situational factors (9–11). This biopsychosocial perspective combines the biological aspects of disease and injury with psychological factors related to injury, patient personality and social factors to give the therapist a way to predict injury and subsequent rehabilitation outcome (11–14). The biological perspective relates to the severity and magnitude of injury on the body. This perspective considers the gravity of injury, likelihood of healing and patient quality of life. Psychological factors include patient behaviors, thoughts and feelings. Does the person have a good attitude, what is the patient's mental health status, does the patient live an active life, is the person accepting of his or her injury, etc. Social factors include the patient's support system(s) and socio-demographics of the injured individual (11–14). Does the patient have a way to get to therapy? Is there someone at home who can help assist the person with therapy? Does the person have a support group that will help he or she deal with the mental and physical aspects of injury?

These biopsychosocial perspectives can act like barriers to healing and rehabilitation success. In 2010, a systematic review was published in the Journal of Manual Therapy, the purpose of which was to discuss barriers to physical therapy in patients undergoing musculoskeletal rehabilitation (15). The authors found a strong level of evidence indicating that having poor fitness and being physically inactive prior to injury lead to decreased treatment success following physical therapy. It was also determined that certain psychological barriers such as high levels of depression, anxiety, or stress and feelings of helplessness were strongly associated with poor adherence to physical therapy treatment programs. Socio-demographic barriers also affected physical therapy participation and success. Individuals with poor family support or an unstable social group were more apt to poorly adhere to the physical therapy program. Biopsychosocial barriers have been shown to directly affect PR planning and success. As such, interventions addressing these barriers and considering these barriers are proven to better patient outcome (16).

## Biopsychosocial Perspective in Canine Physical Rehabilitation

It is interesting and important to consider these biopsychosocial perspectives as they relate to our canine physical rehabilitation patients. When considering spinal cord injury specifically, the biological perspective in the canine patient is well-established. It is common knowledge that the presence or absence of limb deep pain sensation or incontinence following spinal cord injury are biological aspects of disease that directly affect both short and long term outcome and quality of life. Also of importance are previous injuries or medical co-morbidities that one patient may have that directly affects his or her ability to be treated. A paraplegic Corgi, who, for years has been clinical for elbow osteoarthritis, will have a different rehabilitation assessment and different rehabilitation plan than a dog who has no orthopedic disease pre-spinal cord injury, for example. As this patient rehabilitates and is forced to bear an increased percentage of weight on his thoracic limbs, his already diseased elbows may suffer as will his therapy success.

Equally important to assessment and outcome should be the psychological state of the canine patient. Canine patients with spinal cord injury many times may require prolonged hospitalization during recovery from surgery and/or initial injury. They suffer a loss of independence as they may require assistance to walk and even urinate. This change in lifestyle results in a need to adapt; this acute loss of physical function causes stress (17, 18). Since personality and the cognitive state of the individual dog is unique, it is expected that this adaptation process would be expressed through their own behavior and demeanor. Breed types, age, sex and physical fitness and the actual disease from which the animal is suffering also contribute to their adaptation process (18). Although overall biological and genetic basis of breed differences in behavior are, as yet, not fully understood (19), clinically, anyone who performed physical rehabilitation on the average, middle-aged non-ambulatory tetraparetic Dachshund as compared to a neurologically identical Doberman pinscher, knows personality, attitude and motivation make an enormous difference in healing, recovery, and PR success. Generally, Dachshunds seem almost bred to deal well with immobility and rehabilitation while many Dobermans are caught in a perpetual “woe is me state.” They tend to be so preoccupied with their injury and newfound immobility that they do not deal mentally with their injuries as well as the neurologically identical Dachshund. Age and physical fitness are also important. A young Shih Tzu who has no other co-morbidities and is used to being held and carried by his owner will appear to handle being recumbent and doted on by a rehabilitation professional better than an independent, elderly Malamute with degenerative joint disease of the elbows and hips. Physical fitness is also important. A 5-year-old Border collie who loves to exercise and is always moving will likely be more willing to effectively participate in rehabilitation exercises as compared to a typically sedentary Bassett Hound. Physical fitness can also play an integral role in motivation. A quiet, introspective collie will need a different approach when it comes to PR than a hyperactive Weimaraner.

Recently behavior and cognitive states in dogs with medical conditions has gained attention. It is reported that dogs with idiopathic epilepsy tend to have behavioral changes before and after the development of epilepsy (20, 21). In both studies, the owners of dogs with idiopathic epilepsy reported behavior changes in the study survey. The changes included fear, anxiety, aggression, and abnormal perception related to the diagnosis of idiopathic epilepsy. Some behavior signs were observed (e.g., fear and anxious behavior signs) irrespective of antiepileptic drugs (AEDs) prescribed. Since prevalence of psychiatric conditions in people with epilepsy is well-known (22), it is likely that similar etiological background may exist in canine patients with idiopathic epilepsy. Separately, Cockburn et al. (23) investigated the cognitive states in Cavalier King Charles Spaniels (CKCSs) with syringomyelia (*n* = 8) compared with CKCSs without the condition (*n* = 13). Dogs with the condition had more a negative affective state (i.e., pessimistic) assessed by one of the cognitive tests (e.g., the judgment bias test) that was not correlated with clinical signs (e.g., scratching). Although more research into the behavioral and cognitive state in dogs with medical conditions is necessary, these previous studies suggest that the effect of disease (such spinal cord injury) may directly alter behavior and cognition ultimately affecting treatment and prognosis. It is critical that the rehabilitation professional consider the patient's personality, behavior, cognitive state, and level of motivation when assessing the patient for PR as it directly affects outcome and expectation.

The social impact of injury is perhaps the hardest aspect of the biopsychosocial perspective to parlay over to the canine patient. That being said, dogs too, are social beings within a family unit. Though dogs do not have societal pressures weighing down on them, they do have a role within the household. Structured routine and predictable schedules help to reduce dog stress. In a household in which the dog holds a position equitable to the human members of the family it may be easier to obtain owner participation and PR buy-in than in a household in which the dog is simply an outdoor lawn ornament. As such, the role dogs play in the social environment within their home must be considered when assessing the patient for PR and predicting rehabilitation outcome and success.

Because most dogs are very valued members of the family unit, attention should be paid to caregiver burden. The definition of caregiver burden in human medicine can apply to veterinary medicine; it is defined as the strain associated with caregiving. This strain comes in two varieties: subjective (emotional) response plus objective (physical) demands of caregiving (24). For many humans this burden lasts as long as providing care is necessary. Caregiver burden worsens as patient's medical condition worsens, as more care is demanded (25). In a 2018 paper by Spitznagela et al. regarding pets and caregiver burden, owners of dogs or cats with serious illness reported greater burden than those with healthy pets. This burden manifested itself as psychosocial distress, including above average levels of stress and clinically meaningful symptoms of depression. Caregiver burden is also directly related to the financial stress of caregiving, caregiver guilt, and fear of what the future holds for their pet following injury or illness (26, 27). Proper PR takes a commitment of time, ability (both physical and emotional) and a financial investment from the owner in order to achieve success. As such, physical rehabilitation may be associated with caregiver burden in the veterinary patient with spinal cord injury. The owners of a newly paraplegic Rottweiler must feel caregiver burden following a veterinary appointment during which they are given at-home PR exercises to perform, shown how to express their pet's urinary bladder and shown how to sling walk their paraplegic dog. They must feel a burden when they are instructed to return for in-hospital PR 3 days per week. They are then faced with the logistics of physically getting their paralyzed sixty-pound dog to the appointment, taking time off work to make this appointment and also budgeting for the cost associated with this appointment. The caregiver also has to deal with the fear, uncertainty and helplessness that comes with seeing their beloved pet immobile, dependent on them for simple life activities such as urination, and walking.

## The Flag System

In an attempt to consider objectively these biopsychosocial barriers as they pertain to assessment and success in human physical therapy following injury, a flag system was created (28). Flags are assigned different colors from red to black. Red flags represent clinical signs associated with serious disease pathology, orange represent psychiatric symptoms of depression, yellow represent expectations for a poor outcome following therapy such as worry, fear, anxiety, and avoidance of certain PT exercises. Blue flags represent those patients with workplace avoidance related to their injury; these persons do not want to return to work for fear they will exacerbate their injury, or that co-workers will be unsupportive. Black flags represent those with health care or insurance issues that are obstacles to PT planning and enrollment (28).

Though it is clear that some of these “flags” cannot directly be applied to veterinary medicine, the idea that patient behavior and cognitive state are integral to the PR assessment, outcome and PR success traverses species. A “flag” system for canines suffering spinal cord injury could be adapted to assess objectively the injured canine patient (Table 1) at the initial PR appointment. Some flags are already in place when it comes to canine spinal cord injury. For example, a lack of deep pain sensation would represent a red flag, orange would represent those patients who appear depressed or lack the motivation to try during physical rehabilitation exercises. Yellow flags could represent those dogs who suffer from underlying behavioral issues such as anxiety or stress or aggression, those dogs whose issues prevent them from effectively participating in the PR experience. There is no obvious veterinary correlation for the blue flag and thus it can be deleted. The black flag would represent those dogs whose owners cannot afford the recommended in hospital physical rehabilitation with a certified rehabilitation professional or those clients unwilling to participate in at home rehabilitation with their immobile pet.

**Table 1:** Veterinary physical rehabilitation adaptation of the “Flags” system utilized in human physical therapy.

**Color flag**	**Description of the dog**
Red	Biological perspective–Poor neurologic status (example, lacking deep pain sensation on examination to the pelvic limbs, tail, and/or perineum)
Orange	Behavioral and cognitive perspective–Patients who appear depressed or lack motivation. Those who do not participate during physical rehabilitation. Those for which there are no obvious ways to provide motivation (example patient is not food motivated)
Yellow	Behavioral and cognitive perspective–Patients who suffer from behavioral issues (separation anxiety, aggression, or destructive behavior) that prevent them from focusing on exercises or participating in PR. The same issues are further compounded by immobility. Meaning, the lack of mobility in patients who have underlying anxiety may lead to further frustration, depression, or poor motivation
Black	Social perspective–Owners lack the financial ability to have physical rehabilitation performed by a rehabilitation professional. Owners are unwilling to perform the necessary at-home PR exercises

The use of a flag system would help give the rehabilitation professional a more objective way to assess his or her patients for PR. It would allow the professional a way to objectively incorporate the biological, but also behavioral, cognitive and social aspects of injury into his or her rehabilitation assessment. It would also force the rehabilitation professional to consider the cognitive state, behavior and also social barriers when formulating a rehab assessment and plan.

## Conclusion

Considering the biopsychosocial perspective has merit when assessing any canine patient for physical rehabilitation. These perspectives may even be of use when creating an in hospital and/or at home physical rehabilitation plan, and ultimately, can be used to predict overall patient outcome and success following injury and subsequent rehabilitation. As future studies exploring the use of physical rehabilitation in the veterinary spinal cord injury patient are designed and conducted, differences in the canine patient biopsychosocial perspective should be considered.

## Author Contributions

Both authors listed have made a substantial, direct and intellectual contribution to the work, and approved it for publication.

## Conflict of Interest

The authors declare that the research was conducted in the absence of any commercial or financial relationships that could be construed as a potential conflict of interest.

## Publisher's Note

All claims expressed in this article are solely those of the authors and do not necessarily represent those of their affiliated organizations, or those of the publisher, the editors and the reviewers. Any product that may be evaluated in this article, or claim that may be made by its manufacturer, is not guaranteed or endorsed by the publisher.

## Footnotes

^1^College of Veterinary Medicine North Carolina State University. (2020). *Canine Spinal Cord Injury Program*. Available online at: https://cvm.ncsu.edu/research/labs/clinical-sciences/canine-spinal-cord-injury/ (accessed August 23, 2020).
